# Supplementary material for: Analysis of a double Poisson model for predicting football results in Euro 2020
Source: PLoS One. 2022 May 19;17(5):e0268511. doi: 10.1371/journal.pone.0268511 (PMC9119507; doi:10.1371/journal.pone.0268511)
Supplement: S1 File — (ZIP) [file pone.0268511.s001.zip › S1_File.pdf]

S1 - Modelling $\mu_{A,B}$

As stated in the manuscript, it will be assumed that each team scores goals according to an independent Poisson Process, so that

$$\text{Number of goals scored by Team A against Team B} \sim \text{Poi}(\mu_{A,B}), \tag{1}$$

where $\mu_{A,B}$ is the expected number of goals.

To make further progress, it is necessary to estimate the values of $\mu_{A,B}$. The small number of relevant games between countries means that treating each of the $\mu_{A,B}$ as independent variables is impossible - there are many pairs of countries that have not played against each other in the last ten years! Thus, it is necessary to reduce the number of free variables.

This reduction is made possible by assigning each team, A, an offensive strength, O_A, and a defensive strength, D_A. Then, $\mu_{A,B}$ is assumed to be a linear function of these variables of O_A and D_B of the form

$$\mu_{A,B}(O_A, D_B) = \alpha O_A D_B + \beta O_A + \gamma D_B + \delta. \tag{2}$$

It is assumed that if $O_A = 0$, then Team A will never score, which means $\mu_{A,B}(0, D_B) = 0$ and hence

$$\gamma = \delta = 0. \tag{3}$$

Furthermore, one can scale

$$O_A \sim \beta \quad \text{and} \quad D_B \sim -\frac{\alpha}{\beta} \tag{4}$$

to reduce Eq (2) to

$$\mu_{A,B}(O_A, D_B) = O_A(1 - D_B). \tag{5}$$

Finally, for convenience, define defensive vulnerability $V_B := 1 - D_B$ so that $\mu_{A,B}$ simply becomes

$$\mu_{A,B}(O_A, V_B) = O_A V_B. \tag{6}$$
