# Supplementary material for: Analysis of a double Poisson model for predicting football results in Euro 2020
Source: PLoS One. 2022 May 19;17(5):e0268511. doi: 10.1371/journal.pone.0268511 (PMC9119507; doi:10.1371/journal.pone.0268511)
Supplement: S2 File — (ZIP) [file pone.0268511.s002.zip › S2_File.pdf]

S2 - Proofs of Theorems 1 and 2

Solution existence

This section provides a proof of Theroem 1 - the conditions that determine whether ℓ has a finite global maximum.

Statement of Theorem 1

Theorem 1 *Define the set of teams to be T. For any subset $S \subseteq T$, define $Q(S)$ to be the set of teams that have played at least one match against at least one of the teams in S. That is*

$$Q(S) = \{A \in T : \exists B \in S \quad s.t. \quad P_{A,B} > 0\}. \tag{1}$$

Moreover, define

$$G_{A,B} := \textit{Total Number of Goals Scored by } A \textit{ against } B, \tag{2}$$

and define $R(S)$ to be the set of teams that at least one of the teams in S has scored against. That is,

$$R(S) = \{B \in T : \exists A \in S \quad s.t. \quad G_{A,B} > 0\}. \tag{3}$$

Then, there exists a finite global maximum of the log-likelihood $\ell(\boldsymbol{O}, \boldsymbol{V})$ if and only if for any non-empty strict subset $S \subset T$,

$$R\left[Q(S)\right] = S \Rightarrow R[Q(S) \cap Q(T/S)] = \emptyset. \tag{4}$$

Variables that can equal 0

A preliminary note is that, if all variables are finite, then

$$O_A = 0 \Rightarrow O_A V_B = \mu_{A,B} = 0 \quad \forall B \quad \text{and} \quad V_B = 0 \Rightarrow O_A V_B = \mu_{A,B} = 0 \quad \forall A. \tag{5}$$

Now, ℓ can be rewritten as

$$\ell(\boldsymbol{O}, \boldsymbol{V}) = \sum_{A,B} -P_{A,B}\mu_{A,B} + G_{A,B}\ln(\mu_{A,B}). \tag{6}$$

Thus, in order for $\ell \neq -\infty$, it is necessary that if any $\mu_{A,B} = 0$, there must be no $\ln(\mu_{A,B})$ terms in ℓ. That is

$$\mu_{A,B} = 0 \Rightarrow G_{A,B} = 0 \tag{7}$$

and so

$$O_A = 0 \Rightarrow (G_{A,B} = 0 \quad \forall B) \Rightarrow f_A = 0 \tag{8}$$

and similarly

$$V_B = 0 \Rightarrow c_B = 0. \tag{9}$$

Conversely, if $f_A = 0$ then ℓ is non-increasing in O_A. Thus, if there exists a finite maximum, it can be freely assumed that there is a finite maximum such that

$$f_A = 0 \Leftrightarrow O_A = 0. \tag{10}$$

Similarly, it can be assumed that

$$c_B = 0 \Leftrightarrow V_B = 0. \tag{11}$$

Thus, define two sets:

$$F := \{A \in T : f_A > 0\} \quad \text{and} \quad C := \{A \in T : c_B > 0\}\,. \tag{12}$$

If either $F = \emptyset$ or $C = \emptyset$, then one has

$$F = C = \emptyset \tag{13}$$

as all $G_{A,B}$ terms are zero. Thus,

$$\mu_{A,B} = 0 \quad \forall A, B \in T \tag{14}$$

and so, in particular, setting all variables to zero gives an optimal solution. Note that this obeys the conditions of Theorem 1 as $R(S) = \emptyset$ for any set $S \subset T$. Thus, it will hereafter be assumed that both F and C are non-empty.

Then, note that

$$A \in F \Rightarrow O_A > 0 \quad \text{and} \quad B \in C \Rightarrow V_B > 0. \tag{15}$$

Restricting the feasible set

Now, from this, it is helpful to note that the problem

$$\max \left\{ \ell(\boldsymbol{O}, \boldsymbol{V}) : \boldsymbol{O}, \boldsymbol{V} \in \Re^{|T|} \;, \boldsymbol{O}, \boldsymbol{V} \geq \boldsymbol{0} \right\} \tag{16}$$

is equivalent to the problem

$$\max \{\; \ell(\boldsymbol{O}, \boldsymbol{V}) : \boldsymbol{O}, \boldsymbol{V} \in \Re^{|T|}, (0 < O_A \leq 1 \quad \forall A \in F), \quad (O_A = 0 \quad \forall A \notin F) ... \\ ... (V_B > 1 \quad \forall B \in C), \quad (V_B = 0 \quad \forall V \notin C) \;\}\,. \tag{17}$$

This follows from the fact that for any scalar $\rho > 0$

$$\ell(\rho \boldsymbol{O}, \frac{1}{\rho} \boldsymbol{V}) = \ell(\boldsymbol{O}, \boldsymbol{V}) \tag{18}$$

and so, in particular, one can define

$$\rho := \min \left\{ \frac{1}{\max(\boldsymbol{O})} \;,\; \min(\{V_B : B \in C\}) \right\} \in (0, \infty) \tag{19}$$

to create a new pair $(\boldsymbol{O}, \boldsymbol{V})$ with the same value of ℓ and the additional conditions in (17) now met. Note that $\rho \in (0, \infty)$ by the assumption that F and C are non-empty. For the remainder of the proof, the conditions in Eq (17) will be assumed, and the new feasible set will be denoted by W.

A contradictory subsequence

Now, suppose that there is no finite positive local maximum for ℓ. Then, there must be a sequence of strengths $((\boldsymbol{O})^m, (\boldsymbol{V})^m) \in W^N$ such that

$$\lim_{m \to \infty} (\ell((\boldsymbol{O})^m, (\boldsymbol{V})^m)) > \ell(\boldsymbol{O}, \boldsymbol{V}) \quad \forall (\boldsymbol{O}, \boldsymbol{V}) \in W. \tag{20}$$

As the sequence $(\boldsymbol{O})^m$ is bounded, and the sequence $(\boldsymbol{V})^m$ is bounded below, the Bolzano-Weierstrass Theorem states there exists a subsequence m_k such that

$$\forall A \in T \quad \lim_{k \to \infty} O_A^{m_k} = O_A^* \in [0, 1] \quad \text{and} \quad \forall B \in T \quad \lim_{k \to \infty} V_B^{m_k} = V_B^* \in [1, \infty]. \tag{21}$$

Thus, for the remainder of this proof, it will be assumed that this subsequence has been used, so that the explict dependence on k is dropped.

A condition on the sequence

With this setup complete, it is now possible to make progress on the proof. Using Eqs (17) and (20), one of the following two conditions must be met:

$$\exists A^* \quad \text{s.t.} \quad O^m_{A^*} \to 0 \text{ as } m \to \infty \quad \text{and } A \notin F \tag{22}$$

$$\exists B^* \quad \text{s.t.} \quad V^m_{B^*} \to \infty \text{ as } m \to \infty. \tag{23}$$

If these both do not hold, then the sequence $((\boldsymbol{O})^m, (\boldsymbol{V})^m)$ is bounded away from 0, and hence

$$(\boldsymbol{O}^*, \boldsymbol{V}^*) \in W. \tag{24}$$

Thus, by continuity of ℓ in W,

$$\lim_{k\to\infty} (\ell((\boldsymbol{O})^m, (\boldsymbol{V})^m)) = \ell(\boldsymbol{O}^*, \boldsymbol{V}^*) \tag{25}$$

which contradicts Eq (20). Thus, one of Eqs (22) and (23) must hold.

A condition on the limits

Note that ℓ can be rewritten as

$$\ell(\boldsymbol{O}, \boldsymbol{V}) = \sum_{A,B} -P_{A,B} O_A V_B + G_{A,B} \ln(O_A V_B). \tag{26}$$

Note further that, for any positive O_A and V_B, elementary calculus gives

$$-P_{A,B} O_A V_B + G_{A,B} \ln(O_A V_B) \leq -G_{A,B} + G_{A,B} \ln\left(\frac{G_{A,B}}{P_{A,B}}\right). \tag{27}$$

Thus, for any $\tilde{A}$ and $\tilde{B}$

$$\begin{aligned} \lim_{m\to\infty} (\ell((\boldsymbol{O})^m, (\boldsymbol{V})^m)) \leq & \lim_{m\to\infty} \left(-P_{\tilde{A},\tilde{B}} O^m_{\tilde{A}} V^m_{\tilde{B}} + G_{\tilde{A},B} \ln(O^m_{\tilde{A}} V^m_{\tilde{B}})\right) \\ & + \sum_{\substack{A,B \\ (A,B)\neq(\tilde{A},\tilde{B})}} \left[-G_{A,B} + G_{A,B} \ln\left(\frac{G_{A,B}}{P_{A,B}}\right)\right]. \end{aligned} \tag{28}$$

Thus, if $P_{\tilde{A},\tilde{B}} > 0$ and

$$\lim_{m\to\infty} O^m_{\tilde{A}} V^m_{\tilde{B}} = \infty, \tag{29}$$

then

$$\lim_{m\to\infty} \left(-P_{\tilde{A},\tilde{B}} O^m_{\tilde{A}} V^m_{\tilde{B}} + G_{\tilde{A},\tilde{B}} \ln(O^m_{\tilde{A}} V^m_{\tilde{B}})\right) = \lim_{x\to\infty} \left(-P_{\tilde{A},\tilde{B}} x + G_{\tilde{A},\tilde{B}} \ln(x)\right) = -\infty \tag{30}$$

and so

$$\lim_{m\to\infty} (\ell((\boldsymbol{O})^m, (\boldsymbol{V})^m)) = -\infty, \tag{31}$$

which is a contradiction. Furthermore, if $G_{A,B} > 0$ and

$$\lim_{m\to\infty} O^m_{\tilde{A}} V^m_{\tilde{B}} = 0, \tag{32}$$

then

$$\lim_{m\to\infty} \left(-P_{\tilde{A},\tilde{B}} O^m_{\tilde{A}} V^m_{\tilde{B}} + G_{\tilde{A},\tilde{B}} \ln(O^m_{\tilde{A}} V^m_{\tilde{B}})\right) = \lim_{x\to 0} \left(-P_{\tilde{A},\tilde{B}} x + G_{\tilde{A},\tilde{B}} \ln(x)\right) = -\infty \tag{33}$$

which is again a contradiction. Thus

$$P_{\tilde{A},\tilde{B}} > 0 \Rightarrow \lim_{m\to\infty} \left(O^m_{\tilde{A}} V^m_{\tilde{B}}\right) \neq \infty \quad \text{and} \quad G_{\tilde{A},\tilde{B}} > 0 \Rightarrow \lim_{m\to\infty} \left(O^m_{\tilde{A}} V^m_{\tilde{B}}\right) \notin \{0, \infty\}, \tag{34}$$

noting that

$$G_{\tilde{A},\tilde{B}} > 0 \Rightarrow P_{\tilde{A},\tilde{B}} > 0. \tag{35}$$

The implications of Eq (34)

Define

$$\mathcal{A} := \left\{ A : \lim_{m\to\infty}(O_A^m) = 0 \right\} \tag{36}$$

and

$$\mathcal{V} := \left\{ B : \lim_{m\to\infty}(V_B^m) = \infty \right\}. \tag{37}$$

From Eq (34), it follows that

$$B \in \mathcal{V} \Rightarrow \forall A \in T,\ \lim_{m\to\infty}(O_A^m V_B^m) = \infty \text{ or } A \in \mathcal{A} \tag{38}$$

$$\Rightarrow \forall A \in T,\ P_{A,B} = 0 \text{ or } A \in \mathcal{A} \tag{39}$$

and so

$$B \in \mathcal{V} \Rightarrow Q(B) \subseteq \mathcal{A}. \tag{40}$$

This can be written as

$$Q(\mathcal{V}) \subseteq \mathcal{A}. \tag{41}$$

Similarly,

$$A \in \mathcal{A} \Rightarrow \forall B \in T,\ G_{A,B} = 0 \text{ or } \lim_{m\to\infty}(V_B^m) = \infty \tag{42}$$

and hence

$$A \in \mathcal{A} \Rightarrow R(A) \subseteq \mathcal{V} \tag{43}$$

which means

$$R(\mathcal{A}) \subseteq \mathcal{V}. \tag{44}$$

Now, by the definition of R, it is clear that for any sets C and D

$$C \subseteq D \Rightarrow R(C) \subseteq R(D) \tag{45}$$

as at least one member of D has scored at least one goal against a team from $R(C)$. Thus, combining Eqs (41) and (44) gives

$$R[Q(\mathcal{V})] \subseteq R(\mathcal{A}) \subseteq \mathcal{V} \tag{46}$$

The sufficiency of condition (4) - three cases

Now, suppose that condition (4) holds. Note that

$$c_B = 0 \Rightarrow V_B^m = 0 \Rightarrow B \notin \mathcal{V} \tag{47}$$

and so

$$B \in \mathcal{V} \Rightarrow c_B > 0. \tag{48}$$

Now, suppose $B \in \mathcal{V}$. Then, Team B has conceded a goal, and this must have been against a member of $Q(\mathcal{V})$, as any team not in this set has not played against B. Thus $B \in R[Q(\mathcal{V})]$. Hence, using this fact alongside Eq (46),

$$\mathcal{V} \subseteq R[Q(\mathcal{V})] \subseteq \mathcal{V}, \tag{49}$$

and so

$$.\mathcal{V} = R[Q(\mathcal{V})]. \tag{50}$$

Thus, by the condition (4), either $\mathcal{V} = \emptyset$, $\mathcal{V} = T$ or $R[Q(\mathcal{V}) \cap Q(T/\mathcal{V})] = \emptyset$. These cases will be shown to lead to contradictions, or to the construction of a finite maximum.

Case 1: $\mathcal{V} = \emptyset$

In this case, the solution sequence is bounded. Moreover, note that Eq 44 gives

$$R(\mathcal{A}) \subseteq \mathcal{V} = \emptyset \tag{51}$$

and so

$$\mathcal{A} \cap F = \emptyset, \tag{52}$$

which means that $(\boldsymbol{O}^*, \boldsymbol{V}^*) \in W$. This is a contradiction to the definition of the sequence $((\boldsymbol{O})^m, (\boldsymbol{V})^m)$ and hence there is a finite global maximum for ℓ.

Case 2: $\mathcal{V} = T$

Suppose $\mathcal{V} = T$. Then, (41) gives

$$Q(T) \subseteq \mathcal{A}. \tag{53}$$

Now, if $Q(T) \subset T$, then there is an A such that $P_{A,B} = 0$ for all B and hence $G_{A,B} = 0$ for all B. Thus, $A \notin R[Q(T)] = R[Q(\mathcal{V})] = \mathcal{V}$, which contradicts the assumption that $\mathcal{V} = T$. Thus,

$$Q(T) = T. \tag{54}$$

As $Q(\mathcal{V}) \subseteq \mathcal{A}$, it follows that

$$T = Q(\mathcal{V}) \subseteq \mathcal{A} \tag{55}$$

and hence (as necessarily $\mathcal{A} \subseteq T$)

$$\mathcal{A} = T. \tag{56}$$

Thus,

$$O_A^m \to 0 \quad \forall A \quad \text{and} \quad V_B^m \to \infty \quad \forall B. \tag{57}$$

However, this condition cannot hold. One can find a subsequence m_k such that $\exists\ A^*$ and B^* such that

$$O_{A^*}^{m_k} = \max_A (O_A^{m_k}) \quad \forall k \quad \text{and} \quad V_{B^*}^{m_k} = \min_B (V_B^{m_k}) \quad \forall k. \tag{58}$$

Note such a subsequence exists as there are only finitely many teams. It can be further assumed that in this subsequence

$$V_{B^*}^{m_k} > 0 \tag{59}$$

as $V_{B^*}^{m_k} \to \infty$. Moreover, either

$$O_{A^*}^{m_k} = 0 \Rightarrow O_A^{m_k} = 0 \quad \forall A \Rightarrow F = \emptyset \tag{60}$$

which has been assumed to be false. Thus,

$$O_{A^*}^{m_k} > 0. \tag{61}$$

Then, there exists a subsequence m_{k_l} such that one of the following two conditions holds:

$$\forall l \quad O_{A^*}^{m_{k_l}} \geq \frac{1}{V_{B^*}^{m_{k_l}}} \tag{62}$$

$$\forall l \quad O_{A^*}^{m_{k_l}} \leq \frac{1}{V_{B^*}^{m_{k_l}}}. \tag{63}$$

Suppose that (62) holds. Then, one can define a new sequence

$$\tilde{O}_A^l := \frac{(O_A^{m_{k_l}})}{O_{A^*}^{m_{k_l}}} \quad \text{and} \quad \tilde{V}_B^l := (V_B^{m_{k_l}}) O_{A^*}^{m_{k_l}} \tag{64}$$

which satisfies, for all l, A and B

$$\tilde{O}^l_A \leq 1 \quad \text{and} \quad \tilde{V}^l_B \geq O^{m_{k_l}}_{A^*} V^{m_{k_l}}_{B^*} \geq 1 \tag{65}$$

and

$$\ell(\tilde{\mathbf{O}}^l, \tilde{\mathbf{V}}^l) = \ell(\mathbf{O}^{m_{k_l}}, \mathbf{V}^{m_{k_l}}). \tag{66}$$

Moreover, note that

$$\tilde{O}^l_{A^*} = 1 \quad \forall l \tag{67}$$

and therefore this new sequence does not satsify (57) and so must either satisfy the condition of Case 3 (as Case 1 leads to a contradiction) or converge in W. If the sequence converges in W, then there is a finite global maximum, and so this case can be ignored.

By scaling with $V^{m_k}_{B^*}$ in the case that (63) holds, a similar conclusion is reached (as now, $\tilde{V}^l_{B^*}$ is fixed at 1).

Case 3: $R[Q(\mathcal{V}) \cap Q(T/\mathcal{V})] = \emptyset$

The only remaining possibility is that $R[Q(\mathcal{V}) \cap Q(T/\mathcal{V})] = \emptyset$. In this case, the teams in $Q(\mathcal{V}) \cap Q(T/\mathcal{V})$ have scored no goals, and hence

$$Q(\mathcal{V}) \cap Q(T/\mathcal{V}) \subseteq T/F. \tag{68}$$

Now, if $Q(\mathcal{V}) = \emptyset$, then

$$\mathcal{V} = R[Q(\mathcal{V})] = \emptyset \tag{69}$$

and so the logic of Case 1 shows that there is no solution. Thus, suppose that $Q(\mathcal{V}) \neq \emptyset$. Note that

$$A \in Q(\mathcal{V}) \cap Q(T/\mathcal{V}) \Rightarrow O^m_A = 0. \tag{70}$$

Using this, it is possible to perform a similar scaling argument to that in Case 2, where O_A is only scaled for $A \in Q(\mathcal{V})$ and V_B is only scaled for $B \in \mathcal{V}$. Then, the overall solution is unchanged as

$$A \notin Q(\mathcal{V}),\ B \in \mathcal{V} \Rightarrow P_{A,B} = G_{A,B} = 0 \tag{71}$$

and

$$A \in Q(\mathcal{V}),\ B \notin \mathcal{V} \Rightarrow P_{A,B} = 0 \text{ or } A \in Q(\mathcal{V}) \cap Q(T/\mathcal{V}) \tag{72}$$

$$\Rightarrow P_{A,B} O^m_A = G_{A,B} = 0, \tag{73}$$

so that each term in ℓ will remain the same. Thus, as in Case 2 (provided $|\mathcal{V}| \geq 1$), it is possible to construct a solution where $|\mathcal{A}| + |\mathcal{V}|$ decreases by at least 1 (note that Case 2 is really a special case of Case 3). Thus, by induction, there is a sequence in W with $\mathcal{V} = \emptyset$ or $Q(\mathcal{V}) = \emptyset$. Hence, by the work in Case 1, there is a finite maximum for ℓ.

The necessity of condition (4)

Suppose now that (4) does not hold, so there is a non-empty subset $S \subset T$ that satisfies

$$R[Q(S)] = S \quad \text{and} \quad R[Q(S) \cap Q(T/S)] \neq \emptyset. \tag{74}$$

Suppose for a contradiction that there is a finite optimal solution (O^*_A, V^*_B). Now, for any $A \notin Q(S)$ and $B \in S$, it is necessary that

$$P_{A,B} = 0 \Rightarrow G_{A,B} = 0. \tag{75}$$

Also, for any $A \in Q(S)$ and $B \notin S$,

$$G_{A,B} = 0. \tag{76}$$

Then, note that ℓ can be rewritten as

$$\ell(\boldsymbol{O}, \boldsymbol{V}) = -\sum_{A,B} P_{A,B} O_A V_B + \ln\left(\prod_{A \in Q(S), B \in S} (O_A V_B)^{G_{A,B}}\right) + \ln\left(\prod_{A \notin Q(S), B \notin S} (O_A V_B)^{G_{A,B}}\right) \tag{77}$$

as all non-zero $G_{A,B}$ have been accounted for. Now, define $\epsilon \in (0,1)$ and

$$\tilde{O}^*_A := \begin{cases} \epsilon O^*_A & \text{if } A \in Q(S) \\ O^*_A & \text{otherwise} \end{cases} \quad \text{and} \quad \tilde{V}^*_B := \begin{cases} \frac{1}{\epsilon} V^*_B & \text{if } B \in S \\ V^*_B & \text{otherwise} \end{cases}. \tag{78}$$

Then, the terms of $\ell(\tilde{\boldsymbol{O}}^*, \tilde{\boldsymbol{V}}^*)$ will be considered in turn. Firstly,

$$\ln\left(\prod_{A \notin Q(S), B \notin S} (\tilde{O}^*_A \tilde{V}^*_B)^{G_{A,B}}\right) = \ln\left(\prod_{A \notin Q(S), B \notin S} (O^*_A V^*_B)^{G_{A,B}}\right) \tag{79}$$

so this term is unchanged and

$$\ln\left(\prod_{A \in Q(S), B \in S} (\tilde{O}^*_A \tilde{V}^*_B)^{G_{A,B}}\right) = \ln\left(\prod_{A \in Q(S), B \in S} (\frac{\epsilon}{\epsilon} O^*_A V^*_B)^{G_{A,B}}\right) \tag{80}$$

$$= \ln\left(\prod_{A \in Q(S), B \in S} (O^*_A V^*_B)^{G_{A,B}}\right) \tag{81}$$

so this is also unchanged. Finally, note that by Eq (75),

$$-\sum_{A,B} P_{A,B} (\tilde{O}^*_A \tilde{V}^*_B) = -\left(\sum_{A \in Q(S), B \in S} + \sum_{A \in Q(S), B \notin S} + \sum_{A \notin Q(S), B \notin S}\right) (P_{A,B} \tilde{O}^*_A \tilde{V}^*_B) \tag{82}$$

$$= -\left(\sum_{A \in Q(S), B \in S} + \epsilon \sum_{A \in Q(S), B \notin S} + \sum_{A \notin Q(S), B \notin S}\right) (P_{A,B} O^*_A V^*_B) \tag{83}$$

which is decreasing in ϵ provided that

$$\sum_{A \in Q(S), B \notin S} (P_{A,B} O^*_A V^*_B) \neq 0. \tag{84}$$

Suppose for a contradiction that

$$\sum_{A \in Q(S), B \notin S} (P_{A,B} O^*_A V^*_B) = 0. \tag{85}$$

Then, as each term in the above sum is non-negative, this means that

$$\forall A \in Q(S),\ B \notin S \quad P_{A,B} O^*_A V^*_B = 0. \tag{86}$$

As seen previously, $O^*_A = 0$ only if $f_A = 0$, while $V^*B = 0$ only if $c_B = 0$ Thus, the above condition is equivalent to

$$\forall A \in Q(S),\ B \notin S \quad P_{A,B} = 0 \text{ or } f_A = 0 \text{ or } c_B = 0 \tag{87}$$

and so

$$\forall A \in Q(S) \cap Q(T/S), \quad f_A = 0 \text{ or } c_B = 0. \tag{88}$$

Thus,

$$\forall A \in Q(S) \cap Q(T/S), \quad G_{A,B} = 0 \tag{89}$$

so

$$R[Q(S) \cap Q(T/S)] = \emptyset, \tag{90}$$

which is a contradiction. Thus, taking ϵ from 1 to 0 (and hence $\tilde{V}_B^* \to \infty$ for $B \in S \neq \emptyset$) increases the value of ℓ along a path joining the supposed maximum with ∞. Thus, it was not a local maximum as required.

Solution uniqueness

This section provides the proof of Theorem 2 - the conditions on the uniqueness of a solution for $\mu_{A,B}$.

Statement of Theorem 2

Theorem 2 *Define*

$$F := \{A \in T : f_A > 0\} \quad \textit{and} \quad C := \{A \in T : c_B > 0\}\,. \tag{91}$$

Suppose that F (and hence C) is non-empty. Then, the values of $\mu_{A,B} = O_A V_B$ are the same at each local maximum if and only if a finite maximum exists and for each non-empty set S,

$$S \subset F \Rightarrow S \subset Q\left[Q(S) \cap C\right] \cap F \tag{92}$$

and for any $B \in T$

$$B \notin F \Rightarrow Q(B) \cap C \neq \emptyset \tag{93}$$

and

$$B \notin C \Rightarrow Q(B) \cap F \neq \emptyset. \tag{94}$$

Two solutions

Suppose that there exist two finite local maxima $(\mathbf{O}, \mathbf{V})$ and $(\tilde{\mathbf{O}}, \tilde{\mathbf{V}})$ and that the conditions of Theorem 2 hold. Suppose that $B \in C$. Hence, as in the proof of Theorem 1, ℓ is non-increasing in V_B. In fact, ℓ is strictly decreasing in V_B unless

$$\forall A \in T \quad P_{A,B} O_A = 0 \tag{95}$$

which (as $O_A = 0$ only if $f_A = 0$ at a maximum) implies

$$\forall A \in T \quad P_{A,B} = 0 \text{ or } f_A = 0 \Rightarrow Q(B) \cap F = \emptyset, \tag{96}$$

which is a contradiction to condition (94) as $B \notin C$. Thus, ℓ is strictly decreasing in V_B for all feasible parameter values and so it must be the case that

$$B \notin C \Leftrightarrow V_B = \tilde{V}_B = 0. \tag{97}$$

Similarly, using condition (93)

$$A \notin F \Leftrightarrow O_A = \tilde{O}_A = 0. \tag{98}$$

Thus, it remains to show uniqueness for $\mu_{A,B}$ such that $A \in F$ and $B \in C$ (as all other $\mu_{A,B}$ are zero). Note that there exist unique vectors $\boldsymbol{\rho}$ and $\boldsymbol{\kappa}$ of positive scales such that

$$O_A = \rho_A \tilde{O}_A \quad \forall A \in F \quad \text{and} \quad V_B = \frac{\tilde{V}_B}{\kappa_B} \quad \forall B \in C. \tag{99}$$

An equation system for the scales

Now, for $A \in F$

$$-\sum_{B\in T} P_{A,B}\tilde{V}_B + \frac{f_A}{\tilde{O}_A} = 0 \Rightarrow -\sum_{B\in T} P_{A,B}\kappa_B V_B + \frac{f_A \rho_A}{O_A} = 0 \Rightarrow \rho_A = \sum_{B\in C} \frac{P_{A,B} V_B O_A}{f_A}\kappa_B \tag{100}$$

as $B \notin C$ means that $V_B = 0$. Thus, there is a linear system for $\boldsymbol{\kappa}$ and $\boldsymbol{\rho}$, given by

$$\boldsymbol{\rho} = \mathbf{M}\boldsymbol{\kappa} \tag{101}$$

where

$$M_{A,B} = \frac{P_{A,B} V_B O_A}{f_A} \quad \forall A \in F \quad \text{and} \quad \forall B \in C. \tag{102}$$

Similarly,

$$-\sum_{A\in T} P_{A,B}\tilde{O}_A + \frac{c_B}{\tilde{V}_B} = 0 \Rightarrow \frac{1}{\kappa_B} = \sum_{A\in F} \frac{P_{A,B} V_B O_A}{c_B \rho_A}. \tag{103}$$

This is a linear system for $\boldsymbol{\rho}^{-1}$ and $\boldsymbol{\kappa}^{-1}$, where for example

$$\boldsymbol{\rho}^{-1}_A := \frac{1}{\rho_A}, \tag{104}$$

given by

$$\boldsymbol{\kappa}^{-1} = \hat{\mathbf{M}}\boldsymbol{\rho}^{-1} \tag{105}$$

where

$$\hat{M}_{A,B} = \frac{P_{B,A} V_A O_B}{c_A} \quad \forall A \in C \quad \text{and} \quad \forall B \in F. \tag{106}$$

Thus, the equations have been reduced to a closed system for $\boldsymbol{\rho}$ and $\boldsymbol{\kappa}$.

An important property of $\mathbf{M}$ and $\hat{\mathbf{M}}$

Note that for any $A \in F$

$$\sum_{B\in C} M_{A,B} = \sum_{B\in C} \frac{P_{A,B} V_B O_A}{f_A} = \sum_{B\in T} \frac{P_{A,B} V_B O_A}{f_A} = \frac{f_A}{f_A} = 1, \tag{107}$$

while similarly, for any $A \in C$

$$\sum_{B\in F} \hat{M}_{A,B} = \sum_{B\in F} \frac{P_{B,A} V_A O_B}{c_A} = 1. \tag{108}$$

Noting that $\boldsymbol{\rho}$ and $\boldsymbol{\kappa}$ are positive, this means that Eq (101) gives, for any $A \in F$

$$\rho_A \in \left[\min(\boldsymbol{\kappa})\left(\sum_{B\in C} M_{A,B}\right), \max(\boldsymbol{\kappa})\left(\sum_{B\in C} M_{A,B}\right)\right] = [\min(\boldsymbol{\kappa}), \max(\boldsymbol{\kappa})] \tag{109}$$

so

$$\min(\boldsymbol{\rho}) \geq \min(\boldsymbol{\kappa}). \tag{110}$$

Similarly, (105) gives that for any $B \in C$

$$\kappa_B^{-1} \in \left[\min(\boldsymbol{\rho}^{-1}), \max(\boldsymbol{\rho}^{-1})\right]. \tag{111}$$

so

$$\min(\boldsymbol{\kappa}) \geq \min(\boldsymbol{\rho}). \tag{112}$$

Thus,

$$\min(\boldsymbol{\kappa}) = \min(\boldsymbol{\rho}) := m. \tag{113}$$

The sufficiency of the conditions of Theorem 2

From this point, one can show that all elements of $\boldsymbol{\kappa}$ and $\boldsymbol{\rho}$ are equal to m. Suppose that $\rho_A = m$ for $A \in F$ and that there exists a B^* such that $\kappa_{B^*} \neq m$ so necessarily $\kappa_{B^*} > m$. Then,

$$m = \rho_A = \sum_{B\in C} M_{A,B}\kappa_B = \sum_{B\in C/\{B^*\}} M_{A,B}\kappa_B + M_{A,B^*}\kappa_{B^*} \leq (1-M_{A,B^*})m + M_{A,B^*}\kappa_{B^*}. \tag{114}$$

Thus,

$$M_{A,B^*}m \leq M_{A,B^*}\kappa_{B^*} \tag{115}$$

which (given $\kappa_{B^*} > m$) can only hold if $M_{A,B^*} = 0$. Noting that for $B^* \in C$ and $A \in F$, O_A, V_B and f_A are all positive, this means

$$M_{A,B^*} = 0 \Rightarrow P_{A,B^*} = 0. \tag{116}$$

Thus, for any $A \in F$

$$\rho_A = m \Rightarrow \kappa_B = m \quad \forall B \in Q(A) \cap C. \tag{117}$$

Similarly, one can also show that for any $B \in C$

$$\kappa_B = m \Rightarrow \rho_A = m \quad \forall A \in Q(B) \cap F. \tag{118}$$

Combining these conditions shows that

$$\rho_A = m \Rightarrow \rho_{\tilde{A}} = m \quad \forall \tilde{A} \in Q\left[Q(A) \cap C\right] \cap F. \tag{119}$$

Noting that, by the condition (92),

$$A \subset Q\left[Q(A) \cap C\right] \cap F \tag{120}$$

one can hence prove by induction that $\rho_A = m$ for all $A \in F$. This follows by defining

$$S_1 = A \quad \text{and} \quad S_{n+1} = Q\left[Q(S_n) \cap C\right] \cap F, \tag{121}$$

so that for any $n \geq 1$, using conditions (92) and (119),

$$|S_n| \geq \max(n, |F|) \quad \text{and} \quad \rho_A = m \quad \forall A \in S_n. \tag{122}$$

Thus, there is an N such that $S_N = F$ which gives the required result. Moreover, one can note that, as each row of $\hat{\mathbf{M}}$ sums to 1,

$$\rho_A = m \quad \forall A \in F \Rightarrow \boldsymbol{\kappa}^{-1} = \hat{\mathbf{M}}\boldsymbol{\rho}^{-1} = m^{-1}\mathbf{1} \tag{123}$$

where $\mathbf{1}$ is a vector of 1s. Thus,

$$\kappa_B = m \quad \forall B \in C. \tag{124}$$

Hence, for each $A \in F$ and $B \in C$,

$$\tilde{O}_A\tilde{V}_B = O_A V_B \frac{m}{m} = O_A V_B \tag{125}$$

so the values of $\mu_{A,B}$ are all uniquely defined as required.

The necessity of the conditions of Theorem 2

Suppose now that (92) does not hold, so that there exists a non-empty $S \subset F$ such that

$$Q\left[Q(S) \cap C\right] \cap F \subseteq S. \tag{126}$$

Suppose for a contradiction that there is a unique solution $(\boldsymbol{O},\boldsymbol{V})$. Then, define a new solution by

$$\tilde{O}_A := \begin{cases} 2O_A & \text{if } A \in S \\ O_A & \text{otherwise} \end{cases} \tag{127}$$

and

$$\tilde{V}_B := \begin{cases} 0.5V_B & \text{if } B \in Q(S) \cap C \\ V_B & \text{otherwise} \end{cases} \tag{128}$$

Note that this changes one of the values of $\mu_{A,B}$ as if

$$Q(S) \cap C = \emptyset \tag{129}$$

then (as $S \neq \emptyset$) one can find $A \in S$ and $B \in C/Q(S)$ (as C is non-empty by assumption) which gives

$$\tilde{\mu}_{A,B} = 2\mu_{A,B} \neq 0, \tag{130}$$

where the non-zero value follows from the fact that $A \in F$ and $B \in C$. Moreover, if

$$Q(S) \cap C \neq \emptyset \tag{131}$$

then one can find $B \in Q(S) \cap C$ and $A \in F/S$ (as $S \subset F$) such that

$$\tilde{\mu}_{A,B} = 0.5\mu_{A,B} \neq 0. \tag{132}$$

However, this transformation does not change the value of ℓ as follows. Note that ℓ depends on $\mu_{A,B}$ if and only if $P_{A,B} \neq 0$. Thus, it is simply necessary to show that

$$\forall A, B \in T \quad P_{A,B} \neq 0 \Rightarrow O_A V_B = \tilde{O}_A \tilde{V}_B. \tag{133}$$

Firstly, note that

$$A \in S, \quad B \in Q(S) \cap C \Rightarrow \tilde{O}_A \tilde{V}_B = (2O_A)(0.5V_B) = O_A V_B \tag{134}$$

and

$$A \notin S, \quad B \notin Q(S) \cap C \Rightarrow \tilde{O}_A \tilde{V}_B = O_A V_B. \tag{135}$$

Moreover, suppose

$$A \in S, \quad B \notin Q(S) \cap C \quad \text{and} \quad P_{A,B} > 0. \tag{136}$$

As $P_{A,B} > 0$, it is necessary that $B \in Q(S)$ and hence, it follows that

$$B \notin C. \tag{137}$$

Suppose that $V_B \neq 0$. Then, ℓ is strictly decreasing in V_B unless

$$P_{A,B} O_A = 0 \quad \forall A \in T. \tag{138}$$

This must hold as otherwise, the solution $(\boldsymbol{O}, \boldsymbol{V})$ is not a local maximum of ℓ. In this case, one can freely change the value of V_B without changing the value of ℓ. This changes one of the values of $\mu_{A,B}$ unless

$$O_A = 0 \quad \forall A \in T. \tag{139}$$

If this holds, then $F = \emptyset$ which is a contradiction, as $S \subset F$. Thus, $V_B = 0$ and hence

$$0 = \tilde{O}_A \tilde{V}_B = O_A V_B \tag{140}$$

as required. Finally, suppose that

$$A \notin S, \quad B \in Q(S) \cap C \quad \text{and} \quad P_{A,B} > 0. \tag{141}$$

As $P_{A,B} > 0$, it follows that $A \in Q\left[Q(S) \cap C\right]$. Hence, as $A \notin S$, by assumption (126),

$$A \notin F. \tag{142}$$

Suppose $O_A > 0$. Then, ℓ is strictly decreasing unless

$$P_{A,B} V_B = 0 \quad \forall B \in T. \tag{143}$$

Again, this must hold to ensure that $(\boldsymbol{O}, \boldsymbol{V})$ is a local maximum and again, if this holds, one can change the value of O_A without changing the value of ℓ. This changes one of the values of $\mu_{A,B}$ (and hence means the solution is non-unique) unless

$$V_B = 0 \quad \forall B \in T. \tag{144}$$

If this is true, then $C = \emptyset$ and so $G_{A,B} = 0$ for any A and B. Thus, $F = \emptyset$ which is again a contradiction.

Thus, $O_A = 0$, meaning that

$$\tilde{O}_A \tilde{V}_B = O_A V_B \tag{145}$$

as required. Thus, the value of ℓ is unchanged and so there is not a unique maximum, giving the required contradiction.

Now, suppose that condition (93) does not hold so there is some A such that

$$A \notin F \quad \text{and} \quad Q(A) \cap C = \emptyset. \tag{146}$$

Suppose there is a unique local maximum $(\boldsymbol{O}, \boldsymbol{V})$. Note that ℓ is non-increasing in O_A and in V_B for any $B \in Q(A) \subseteq T/C$. Thus, in particular, setting the values of V_B to zero for $B \in Q(A)$ will not decrease ℓ and so, by uniqueness, one must have

$$V_B = 0 \quad \forall B \in Q(A). \tag{147}$$

However, then, ℓ is independent of the value of O_A and so it can be changed to any real number. This changes one of the $\mu_{A,B}$ unless

$$V_B = 0 \quad \forall B \in T. \tag{148}$$

If this is true, then, as before, $C = F = \emptyset$ which contradicts the assumption made about these sets. Thus, there must be a non-zero value of V_B and hence, the solution is non-unique for the $\mu_{A,B}$ as required.

An identical argument holds for the case when condition (94) does not hold (indeed, one can simply consider taking the transpose of G) and hence the proof is complete.

A note on the case $F = C = \emptyset$

Note that if both F and C are empty, then

$$\ell(\boldsymbol{O}, \boldsymbol{V}) = -\sum_{A,B} \mu_{A,B} P_{A,B} \tag{149}$$

and so is maximised when

$$\mu_{A,B}P_{A,B} = 0 \quad \forall A, B. \tag{150}$$

Thus, if each $P_{A,B} > 0$ for $A \neq B$ then there is unique maximum at $\mu_{A,B} = 0$ for each $A \neq B$. Otherwise, if $P_{A,B} = 0$ for some $A \neq B$ then note that the solution

$$O_A = x \quad O_C = 0 \quad \forall C \neq A \tag{151}$$

and

$$V_B = y \quad V_C = 0 \quad \forall C \neq B \tag{152}$$

gives a maximum for any values of x and y, so there is not a unique solution.
