# Supplementary material for: Analysis of a double Poisson model for predicting football results in Euro 2020
Source: PLoS One. 2022 May 19;17(5):e0268511. doi: 10.1371/journal.pone.0268511 (PMC9119507; doi:10.1371/journal.pone.0268511)
Supplement: S3 File — This presents the algorithms for checking existence and uniqueness and proves that they give the correct results. (ZIP) [file pone.0268511.s003.zip › S3_File.pdf]

S3 - Efficient checking of existence and uniqueness of solutions

The conditions given in Theorem 1 and Theorem 2 for the existence and uniqueness of solution are, as written, computationally expensive to check (for the set of 55 European football teams, there are of course 2^{55} possible subsets!). However, it is possible to check these conditions much more quickly, as shown below

Checking for existence

Note that

$$R[Q(A \cup B)] = R[Q(A) \cup Q(B)] = R[Q(A)] \cup R[Q(B)] \quad (1)$$

so that

$$R[Q(S)] = S \Rightarrow A \cup R[Q(A)] \subseteq S \quad \forall A \subseteq S. \quad (2)$$

Thus, one can check the condition using the following algorithm which attempts to construct a set S

Algorithm 1 *One can check for existence as follows:*

1) *Initialise with a set of possible teams,* $K = T$.
2) *If* $K = \emptyset$*, the algorithm terminates and the existence condition holds. Otherwise, find* $A \in K$*, define* $S := \{A\}$ *and calculate* $R[Q(S)]$.
3) *If* $S = T$*, then set* $K := K/\{A\}$ *and return to 2.*
4) *If* $R[Q(S)] \subset S$*, then set* $K := K/\{A\}$ *and return to 2.*
5) *If* $S = R[Q(S)]$*, then calculate* $R[Q(S) \cap Q(T/S)]$*. If this is empty then set* $K := K/\{A\}$ *and return to 2. Otherwise, terminate the algorithm as the existence condition does not hold.*
6) *Set* $S := S \cup R[Q(S)]$*, recalculate* $R[Q(S)]$ *and return to 3.*

Proposition 1 *Algorithm 1 returns the correct answer*

Proof: Firstly, it is clear that Algorithm 1 only returns non-existence if it constructs a set that proves non-existence. Thus, it suffices to show that the algorithm always returns non-existence if no solution exists. Hence, suppose that there is a set S that contradicts the existence condition. Then, as

$$R[Q(S) \cup Q(T/S)] \neq \emptyset, \quad (3)$$

there exists an $B \in T$ such that

$$B \in Q(S) \cup Q(T/S) \quad (4)$$

and $R(B) \neq \emptyset$. Furthermore,

$$B \in Q(S) \Rightarrow \quad \exists A \in S \quad \text{s.t.} \quad B \in Q(A). \quad (5)$$

Now, Algorithm 1 recursively calculates

$$S_1 = \{A\} \quad \text{and} \quad S_{n+1} = S_n \cup R[Q(S_n)]. \quad (6)$$

By Eq (2), each S_n satisfies $S_n \subseteq S$. As $S_n \subseteq S_{n+1}$ and S is finite, there exists an N such that

$$S_N = S_{N+1} = S_N \cup R[Q(S_N)]. \quad (7)$$

Thus, in particular

$$R[Q(S_N)] \subseteq S_N. \tag{8}$$

Suppose that

$$K \in S_N / R[Q(S_N)]. \tag{9}$$

Then, Team K must have conceded zero goals as this condition means

$$G_{L,K} = 0 \quad \forall L \in Q(S_N), \tag{10}$$

while $G_{L,K} = 0$ always holds for $L \notin Q(K) \subseteq Q(S_N)$. Hence, $K \notin R[Q(S)]$, which is a contradiction as then $S \neq R[Q(S)]$. Thus, it must be the case that

$$R[Q(S_N)] = S_N. \tag{11}$$

This set will have been found by the algorithm when it used A in step 2. Thus, the algorithm returns non-existence unless

$$R[Q(S_N) \cup Q(T/S_N)] = \emptyset. \tag{12}$$

However, note that $B \in Q(A) \subseteq Q(S_N)$ and $B \in Q(T/S) \subseteq Q(T/S_N)$ so that

$$\emptyset \neq R(B) \subseteq R[Q(S_N) \cup Q(T/S_N)] \tag{13}$$

and so

$$R[Q(S_N) \cup Q(T/S_N)] \neq \emptyset \tag{14}$$

which means the algorithm returns the correct answer as required.

Checking for uniqueness

A similar algorithm can be used to check for the uniqueness of a solution.

Algorithm 2 *One can check for uniqueness as follows:*

1) *Calculate the sets C, F. If F is empty, then terminate the algorithm with uniqueness undetermined.*
2) *For each $A \in T/C$, check whether*

$$Q(A) \cap F = \emptyset. \tag{15}$$

If this holds for some $A \in T/C$ then terminate as the uniqueness condition does not hold.
3) *For each $A \in T/F$, check whether*

$$Q(A) \cap C = \emptyset. \tag{16}$$

If this holds for some $A \in T/F$ then terminate as the uniqueness condition does not hold.
4) *Define $K = F$.*
5) *Choose any $A \in K$ and define $S := \{A\}$.*
6) *Calculate $S' := S \cup (Q[Q(S) \cap C] \cap F)$.*
7) *If $S' = F$ then define $K = K/\{A\}$ and return to 4. Otherwise, if $S = S'$, then the algorithm terminates as the uniqueness condition does not hold. Otherwise, redefine $S = S'$ and return to 5.*

Proposition 2 *This algorithm returns the correct answer if F is non-empty.*

Proof: Recall the conditions in Theorem 2 were that F was non-empty and

$$S \subset F \Rightarrow S \subset Q\left[Q(S) \cap C\right] \cap F \tag{17}$$

and for any $B \in T$

$$B \notin F \Rightarrow Q(B) \cap C \neq \emptyset \tag{18}$$

and

$$B \notin C \Rightarrow Q(B) \cap F \neq .\emptyset \tag{19}$$

The conditions (18) and (19) are checked directly by the algorithm.

Now, suppose there is a non-empty set S such that $S \subseteq Q[Q(S) \cap C] \cap F$. Then find $A \in S$, and define

$$S_1 = \{A\} \quad \text{and} \quad S_{n+1} = S_n \cup (Q[Q(S_n) \cap C] \cap F) \tag{20}$$

so that as each $S_n \subseteq S_{n+1} \subseteq S$, eventually

$$S_N = S_{N+1} = S_N \cup (Q[Q(S_N) \cap C] \cap F), \tag{21}$$

which means that

$$Q[Q(S_N) \cap C] \cap F \subseteq S_N. \tag{22}$$

Moreover, note that $S_N \subseteq S \subset F$, so the algorithm will terminate with non-existence in this case.
Now, suppose that the algorithm terminates with non-existence. Then, there exists some non-empty $S \subset F$ such that

$$S \cup (Q[Q(S) \cap C] \cap F) = S \tag{23}$$

which means

$$Q[Q(S) \cap C] \cap F \subseteq S \tag{24}$$

and so the uniqueness condition does not hold. Thus, the algorithm returns the correct answer.
