# Supplementary material for: Analysis of a double Poisson model for predicting football results in Euro 2020
Source: PLoS One. 2022 May 19;17(5):e0268511. doi: 10.1371/journal.pone.0268511 (PMC9119507; doi:10.1371/journal.pone.0268511)
Supplement: Attachment — Submitted filename: Response To Reviewers.pdf [file pone.0268511.s004.pdf]

Dear Dr Demidenko,

Thank you for your comments on our manuscript. We have written below our responses to each of the points raised.

Sincerely, on behalf of all the authors

Matthew Penn

Main Comments

1. Interpretation of OA and VB is arbitrary – they are just factors. Can't we interpret these quantities as factors contributing to playing home versus guest match? The problem with of your interpretation, although plausible, should be mentioned.

We have clarified that OA and VB are independent of which team is playing at home or away. We have provided further justification for our interpretation, while also acknowledging the possibility for different interpretations to be made.

2. I believe that there is even a simple model to derive the teams standing I want to share with you using the average of the score difference for each team. Indeed, let rA denote the standing/rank of team A and dAB denote the difference in score A played against B, that is, if the score was 1:3 we have dAB = -2. The major assumption made is that dAB is proportional to the difference in teams standing, dAB = a(rA – rB) + epsAB where a>0 is the scale parameter and epsAB is the error term with zero mean and constant variance. In fact, we may forget about a and simply rewrite the model as dAB = rA – rB + epsAB. Indeed, after rA derived we can make the transformation (rA – min)/(max-min). The above is a linear model. Assuming that ∑ rA = 0 we arrive at the simple solution rA = ∑BdAB /nA where nA is the number of matches played by team A. This model has advantages: (a) the solution is easy to interpret, (b) unlike your model there is no technical issues such as existence and uniqueness (say, that all teams played against each other – then you PAB = const). (c) This model uses all scores while you use only the total number of scores. Disadvantage is that eps does not have a normal distribution. I believe that the comparison of the linear with the Poisson model to predict standings would be of great interest and it would strengthen your paper.

We have added in a subsection to compare our model to the linear model described above. We have shown how to derive rankings from our model, and plotted them against the rankings from the linear model in our new Figure 12. We have also calculated the mean error (in goal difference) between the predictions from each model and the actual results of Euro 2020. We have highlighted evidence that the models are similar, but that the extra information used by the Poisson model helps it to give better predictions.

3. Another probabilistic model for ranking the team is suggested in Demidenko E (2020). Advanced Statistics with Applications in R. New York: Wiley.
We have added this as a reference, referring to it in the introduction.

Editorial Comments

1. Refer in the introduction to “football/soccer” at least once because many readers are from US.
We have added in a reference to soccer in the introduction.

2. Use “p-value” not “p value.”
We have changed this.

3. For presentation purpose I’d derive equation (3).
We have added the derivation of Equation 3 into the main manuscript. We have hence removed it from the supplementary material file S1.
