# Supplementary material for: Analysis of a double Poisson model for predicting football results in Euro 2020
Source: PLoS One. 2022 May 19;17(5):e0268511. doi: 10.1371/journal.pone.0268511 (PMC9119507; doi:10.1371/journal.pone.0268511)
Supplement: Attachment — Submitted filename: Review-PONE-D-22-01098R1.pdf [file pone.0268511.s005.pdf]

Review of:

[Manuscript PONE-D-22-01098R1

Title: Analysis of a double Poisson model for predicting football results in Euro 2020

Authors: Matthew J. Penn, Christl A. Donnelly]

I have read the paper with interest.

The manuscript topic is of interest for “PLOS ONE”.

The authors offer a work that potentially can improve our knowledge about the topic of interest. I suggest that the authors consider a revision of their work along the following suggestions and questions.

Suggestions and Questions:

1- It is recommended to start the abstract with general information about the topic.

2- It is suggested to discuss more about the findings of this study in the abstract.

3- It is recommended to mention about the applications of this study at the end of abstract:

The findings of this study can help for better understanding of …

4- I strongly recommend the authors to add one paragraph discussing the difference between their work and the previously performed studies in literature. In other words, what is the novelty of this work? I offer the authors to revise the abstract and introduction in order to incorporate the novelty of their work. This change motivates the readers of “PLOS ONE” to study this work with interest.

5- It is suggested to add a figure in Section 1, which presents the general sketch of the problem under study.

6- It is recommended to include a paragraph at the end of introduction to present the steps of the work like:

First, the methods are presented. Then,…

7- What are the advantages and disadvantages of this study? I recommend the authors to highlight this topic.

8- What are the limitations of this study? I recommend the authors to highlight this topic.

9- The boxes around the legends in all the plots should be removed.

10- “Where” should be replaced by “where” for definition of parameters or variables after each equation.

11- It is recommended to add minor ticks (or intervals) on horizontal and vertical axis of all the plots.

12- The title for last section should be changed to “Summary and Conclusions”.

13- It is recommended to show the main remarks of this study in terms of bullets in last section (Summary and Conclusions).

14- It is suggested to add a nomenclature (including alphabetic letters, Greek letters, subscripts, and superscripts).

15- It is recommended to keep the main governing equations in the text of manuscript and move the rest of equations to an appendix.
