# Supplementary material for: Analysis of a double Poisson model for predicting football results in Euro 2020
Source: PLoS One. 2022 May 19;17(5):e0268511. doi: 10.1371/journal.pone.0268511 (PMC9119507; doi:10.1371/journal.pone.0268511)
Supplement: Attachment — Submitted filename: ResponseToReviewers.pdf [file pone.0268511.s006.pdf]

Dear Reviewer

We have received your comments on our manuscript. We have written below our responses to each of the points raised.

Yours faithfully, on behalf of all the authors

Matthew Penn

**Main Comments**

**1- It is recommended to start the abstract with general information about the topic.**
We have rewritten the abstract to start with some background information on the double Poisson model.

**2- It is suggested to discuss more about the findings of this study in the abstract.**
We have added in some of the findings of this study into the abstract.

**3- It is recommended to mention about the applications of this study at the end of abstract:**
**The findings of this study can help for better understanding of …**
This has been added to the end of the abstract.

**4- I strongly recommend the authors to add one paragraph discussing the difference between their work and the previously performed studies in literature. In other words, what is the novelty of this work? I offer the authors to revise the abstract and introduction in order to incorporate the novelty of their work. This change motivates the readers of “PLOS ONE” to study this work with interest.**
The previous draft contained a paragraph starting “The main novel contribution to this work is...” We have added in the word “novel” to the second sentence of this paragraph to emphasise that the whole paragraph is concerned with the novelty of the study. We have also highlighted the novelty in the abstract.

**5- It is suggested to add a figure in Section 1, which presents the general sketch of the problem under study.**
We have added in this figure.

**6- It is recommended to include a paragraph at the end of introduction to present the steps of the work like:**
**First, the methods are presented. Then,…**
This paragraph has been added into the introduction.

**7- What are the advantages and disadvantages of this study? I recommend the authors to highlight this topic.**
A paragraph discussing the advantages and disadvantages has been added.

**8- What are the limitations of this study? I recommend the authors to highlight this topic.**

The limitations are highlighted in the new paragraph discussing the advantages and disadvantages.

**9- The boxes around the legends in all the plots should be removed.**
The boxes around legends have been removed.

**10- “Where” should be replaced by “where” for definition of parameters or variables after each equation.**
The authors performed a case sensitive search in the main text, and all supplementary material for the word “Where” and did not find any occurrences. However, they are happy to change any of these that have been missed.

**11- It is recommended to add minor ticks (or intervals) on horizontal and vertical axis of all the plots.**
These have been added to all of the plots.

**12- The title for last section should be changed to “Summary and Conclusions”.**
The title for the final section has been changed.

**13- It is recommended to show the main remarks of this study in terms of bullets in last section (Summary and Conclusions).**
The previous conclusion has been moved to a section entitled “Discussion”. The section “Summary and Conclusions” now contains bullets.

**14- It is suggested to add a nomenclature (including alphabetic letters, Greek letters, subscripts, and superscripts).**
A nomenclature has been added.

**15- It is recommended to keep the main governing equations in the text of manuscript and move the rest of equations to an appendix.**
The previous review of this manuscript (by the editor, Eugene Demidenko) requested that the equations in the derivation were placed in the main text. The authors therefore query this comment to the editor - the derivation has been currently left in the main text but can be moved if required.
